# Various Profiles of *tet* Genes Addition to *tet*(X) in *Riemerella anatipestifer* Isolates From Ducks in China

**DOI:** 10.3389/fmicb.2018.00585

**Published:** 2018-03-27

**Authors:** De-Kang Zhu, Hong-Yan Luo, Ma-Feng Liu, Xin-Xin Zhao, Ren-Yong Jia, Shun Chen, Kun-Feng Sun, Qiao Yang, Ying Wu, Xiao-Yue Chen, An-Chun Cheng, Ming-Shu Wang

**Affiliations:** ^1^Research Center of Avian Diseases, College of Veterinary Medicine, Sichuan Agricultural University, Chengdu, China; ^2^Key Laboratory of Animal Disease and Human Health of Sichuan Province, Chengdu, China; ^3^Institute of Preventive Veterinary Medicine, Sichuan Agricultural University, Chengdu, China

**Keywords:** *Riemerella anatipestifer*, tetracycline resistance, resistance gene, PCR, mosaic gene

## Abstract

To investigate tetracycline resistance and resistant genotype in *Riemerella anatipestifer*, the tetracycline susceptibility of 212 *R. anatipestifer* isolates from China between 2011 and 2017 was tested. The results showed that 192 of 212 (90.6%) *R. anatipestifer* isolates exhibited resistance to tetracycline (the MICs ranged from 4 to 256 μg/ml). The results of PCR detection showed that, 170 of 212 (80.2%) *R. anatipestifer* isolates possessed the *tet*(X) gene. Other genes, including *tet*(A), *tet*(M), *tet*(Q), *tet*(O), *tet*(B), and *tet*(O/W/32/O), were found at frequencies of 20.8, 4.7, 1.4, 0.9, 0.9, and 0.5%, respectively. However, *tet*(C), *tet*(E), *tet*(G), *tet*(K), and *tet*(W) were not detected in any isolate. In these *tet* gene positive strains, 31 (14.6%), 2 (0.9%), 5 (2.4%), 1 (0.5%), 3 (1.4%) were detected containing *tet*(A)/*tet*(X), *tet*(M)/*tet*(O), *tet*(M)/*tet*(X), *tet*(O)/*tet*(X), and *tet*(Q)/*tet*(X) simultaneously, respectively. One isolates, R131, unexpectedly contained three *tet* genes, i.e., *tet*(M), *tet*(O), and *tet*(X). Sequence analysis of the *tet* gene ORFs cloned from *R. anatipestifer* isolates confirmed that *tet*(A), *tet*(B), *tet*(M), *tet*(O), *tet*(Q) and an unusual mosaic *tet* gene *tet*(O/W/32/O) were present in *R. anatipestifer*. The MIC results of *R. anatipestifer* ATCC 11845 transconjugants carrying *tet*(A), *tet*(B), *tet*(M), *tet*(O), *tet*(O/W/32/O), *tet*(Q), and *tet*(X) genes exhibited tetracycline resistance with MIC values ranging from 4 to 64 μg/ml. Additionally, the *tet*(X) gene could transfer into susceptible strain via natural transformation (transformation frequencies of ~10^−6^). In conclusion, the *tet*(A), *tet*(B), *tet*(M), *tet*(O), *tet*(O/W/32/O), *tet*(Q), and *tet*(X) genes were found and conferred tetracycline resistance in *R. anatipestifer* isolates. Moreover, the *tet*(X) is the main mechanism of tetracycline resistance in *R. anatipestifer* isolates. To our knowledge, this is the first report of *tet*(A), *tet*(B), *tet*(M), *tet*(O), *tet*(Q), and mosaic gene *tet*(O/W/32/O) in *R. anatipestifer*.

## Introduction

Tetracyclines are one of the cheapest broad-spectrum antibacterial agents, with activity against a wide range of host, including aerobic, anaerobic, gram-positive and gram-negative bacteria (Chopra and Roberts, 2001; Roberts, 2003). The antibiotic activity of tetracycline exerts through targeting the 30S ribosome subunit, resulting in inhibition of protein synthesis (Walsh, 2003). Due to overuse of tetracycline antibiotics in clinics, tetracycline-resistant strains and tetracycline resistance genes (*tet*) have occurred in bacteria with increasing frequency (Chopra and Roberts, 2001; Zhang et al., 2012). The major mechanisms of tetracycline resistance include: active efflux pumps, ribosomal protection and enzymatic modification (Thaker et al., 2010). For most bacteria, the predominant mechanisms of tetracycline resistance are active efflux pumps and ribosomal protection proteins. Currently, there are 59 different genes found in various genera of bacteria conferring tetracycline resistance, including: efflux genes (*n* = 33), ribosomal protection genes (*n* = 12) and enzymatic inactivation genes (*n* = 13) (http://faculty.washington.edu/marilynr/). In addition, an unknown gene *tet*(U) was reported to be located on the pKq10 plasmid in *Enterococcus faecium* (Ridenhour et al., 1996).

*R. anatipestifer* is an avian pathogen found worldwide, primarily infecting domestic ducks, geese, and turkeys, and results in characterized serositis and septicaemia (Ruiz and Sandhu, 2013). Tetracycline is widely used for the prevention and treatment of pathogen infections and the resistance phenomenon was quite serious in the avian industry (Zhang et al., 2012; Zhong et al., 2013). However, little is known about the mechanisms of tetracycline resistance in *R*. *anatipestifer*, except that the *tet*(X) gene was reported to be located on the pRA0511 plasmid in *R*. *anatipestifer* (Chen et al., 2010), and the resistance pump gene *tet*(C) was reported to be the main mechanism of tetracycline resistance in *R*. *anatipestifer* isolates (Zhong et al., 2013).

To determine the tetracycline resistance mechanisms of *R*. *anatipestifer*, we investigated the tetracycline susceptibility and the prevalence of 12 *tet* genes of 212 *R*. *anatipestifer* field isolates. In this study, we first reported that *tet*(A), *tet*(B), *tet*(O), *tet*(O/W/32/O), *tet*(Q) in *R*. *anatipestifer* and their resistant function were confirmed by transferring into tetracycline-susceptible *R*. *anatipestifer* ATCC 11845.

## Materials and methods

### Bacteria, plasmids, and growth conditions

The bacteria and plasmids used in this study are listed in Table S1. The *R. anatipestifer* field isolates were isolated from 58 large-scale duck farms in different regions of China between 2011 and 2017, including Sichuan, Jiangsu, Guizhou, Anhui, Guangdong, Chongqing, Guangxi, Hainan, Jiling, Henan and Beijing provinces. Under sterile conditions, the brains, hearts or livers were collected from infected or died ducks, and samples were streak-inoculated on tryptic soybean agar plates (TSA, Oxoid Ltd, Basingstoke, Hampshire, England) supplementary with 10% sheep-blood. A single colony from one duck was purified and cultured repeatedly. A total of 212 isolates were identified as *R. anatipestifer* by PCR amplifying 16S rRNA and sequencing and biochemical analyses. *R. anatipestifer* strains were cultured at 37°C in GC broth (GCB) or on GC agar (GCA) plates (Liu et al., 2017) when prepared for natural transformation, and were grown at 37°C in tryptic soybean broth (TSB; Oxoid Ltd, Basingstoke, Hampshire, England) or on TSA plates when prepared for susceptibility testing. *Escherichia coli* (*E. coli*) strains DH5α and S17-1 were grown at 37°C in Luria-Bertani (LB; Oxoid Ltd, Basingstoke, Hampshire, England) broth or on LB agar. When required, antibiotics were added as follows: 5 μg/ml tetracycline (TET); 2 μg/ml cefoxitin (FOX); 40 μg/ml kanamycin (KAN) or 100 μg/ml ampicillin (AMP). All antibiotics were obtained from Dalian Meilun Biotech Co., Ltd. (Dalian, China).

### Tetracycline susceptibility testing

The 212 isolates were tested for tetracycline susceptibility in TSB. The micro-dilution method was used to determine the minimal inhibitory concentration (MIC) in 96-well microtiter-plates (Corning, NY, USA) according to Clinical and Laboratory Standards Institute (CLSI) guideline specific for bacteria isolated from animals (CLSI, 2013). The final concentrations of tetracycline ranged from 0.25 to 512 μg/ml. The concentration of the bacterial inoculum was about 10^6^ CFU/ml (100 μl/well). The inoculated micro-plates were incubated at 37°C for 24 h. *E. coli* ATCC 25922 was used as a quality control strain. The experiments were repeated at least three times. Due to the lack of CLSI-approved tetracycline breakpoints applicable to *R. anatipestifer* (Nhung et al., 2017), and moreover, the tetracycline MIC of reference strain *R. anatipestifer* ATCC 11845 was only 0.25 μg/ml, the *R. anatipestifer* isolates were tentatively classified as resistance to the tetracycline on the basis of the CLSI-approved criteria for *Streptococcus* spp. (CLSI, 2016), i.e., strains with an MIC of tetracycline of ≤1 μg/ml were considered susceptible, 2 μg/ml as intermediate, and ≥4 μg/ml as resistant.

### Detection of the *tet* genes in *R. anatipestifer* field isolates

The PCR templates were prepared by treating the bacteria with lysis buffer (1% Triton, 20 mM Tris-HCl, pH 8.0; 2 mM EDTA, pH 8.0) and boiled for 10 min. The presence of the tetracycline resistance genes (*tet*) characterized previously in other bacteria were determined in all *R. anatipestifer* field isolates by PCR (Ng et al., 2001; Wu et al., 2010). These detected genes included 6 efflux pump genes [*tet*(A), *tet*(B), *tet*(C), *tet*(E), *tet*(G), *tet*(K)], 4 ribosomal protection genes [*tet*(M), *tet*(O), *tet*(Q), *tet*(W)], enzymatic inactivation gene *tet*(X), and a mosaic tetracycline resistance gene *tet*(O/W/32/O). The primers and protocols described previously (Ng et al., 2001; Ghosh et al., 2009; Wu et al., 2010; Koo and Woo, 2011; Palmieri et al., 2012) are listed in Table S2. The *R. anatipestifer* CH-2 containing *tet*(X) was used as positive control. Since the absence of available positive controls for other *tet* genes, all PCR products were size-confirmed by electrophoresis on a 1.5% agarose gel. All amplicons of *tet*(B), *tet*(M), *tet*(O), *tet*(O//W/32/O), and *tet*(Q) from *R. anatipestifer* isolates were verified by sequencing (BGI Tech Solutions Co., Ltd. Shenzhen, China), while only 12 amplicons of *tet*(A) were sequenced.

### Cloning, sequencing, and sequence analysis of *tet* gene ORFs

The ORF of *tet*(X) gene was amplified by PCR from *R. anatipestifer* CH-2 using the primer sets *tet*(X)-F2/*tet*(X)-R2, listed in Table 1. At the same time, the ORFs of *tet*(A), *tet*(B), *tet*(M), *tet*(O), *tet*(O/W/32/O), and *tet*(Q) gene were also amplified from *tet*-positive isolates confirmed above using the additional primers that were designed based on the previously described *tet* sequences in other bacteria (Table 1). The number of amplified ORFs were 6, 2, 5, 2, 1, and 3 for *tet*(A), *tet*(B), *tet*(M), *tet*(O), *tet*(O/W/32/O), and *tet*(Q) genes, respectively. The purified amplicons were sequenced and digested with restriction enzymes, and then ligated to the same digested shuttle vector pLMF03 (Liu et al., 2016). Then the corrected recombinant plasmids were introduced into *E. coli* S17-1. These sequences were analyzed by BLAST in NCBI.

**Table 1:** Primers used for cloning the ORFs of tetracycline resistance genes.

**Genes**	**Primers**	**PCR primer sequences (5′−3′)**	**Size (bp)**	**GenBank accession No**.	**Location**	**Source of plasmid and reference**
*tet*(A)	*tet*(A)-F2	CATGCCATGGGTGAAACCCAACAGACCCCTG	1,200	X61367	Tn1721	Allmeier et al., 1992
	*tet*(A)-R2	CCGCTCGAGTCAGCGATCGGCTCGTTG				
*tet*(B)	*tet*(B)-F2	TGCTCTAGAATGAATAGTTCGACAAAGATCGCATTG	1,206	J01830	Tn10	Hillen and Schollmeier, 1983
	*tet*(B)-R2	CCGCTCGAGCTAAGCACTTGTCTCC				
*tet*(M)	*tet*(M)-F2	CATGCCATGGATGAAAATTATTAATATTGGAGTTTTAGC	1,920	X90939	Tn5251	Provvedi et al., 1996
	*tet*(M)-R2	CCGCTCGAGCTAAGTTATTTTATTGAACATATATCG				
*tet*(O)	*tet*(O)-F2	CATGCCATGGATGAAAATAATTAACTTAGGCATTC	1,920	Y07780	Chromosome	Widdowson et al., 1996
	*tet*(O)-R2	CCGCTCGAGTTAAGCTAACTTGTGG				
*tet*(O/W/32/O)	*tet*(O/W)-F2	CATGCCATGGATGAAAATAATTAACTTAGGCATTCTG	1,920	FR823304	ICESsu3245	Palmieri et al., 2012
	*tet*(O/W)-R2	CCGCTCGAGTTAAGCTAACTTGTGGAAC				
*tet*(Q)	*tet*(Q)-F2	CATGCCATGGGTGCGTTTCGACAATGCATCTATTG	1,974	Z21523	Chromosome	Lépine et al., 1993
	*tet*(Q)-R2	CCGCTCGAGTTATTTTGATGACATTGATTTTTGGAAC				
*tet*(X)	*tet*(X)-F2	CATGCCATGGATGACAATGCGAATAGATACAG	1,167	AGC41071	Chromosome	This study
	*tet*(X)-R2	CCGCTCGAGTTATACATTTAACAATTGCTG				

### Transfer experiment

The correct recombinant plasmids were transferred into *R. anatipestifer* ATCC 11845 by conjugative transfer as previously described (Neela et al., 2009; Luo et al., 2015). Briefly, the *E. coli* S17-1 containing recombinant plasmid was served as donor strain, while the reference strain *R. anatipestifer* ATCC 11845 was served as recipient strain, which does not carry any *tet* genes and is sensitive to tetracycline (Wang et al., 2012). Log-phase donor and recipient strains were mixed in 10 mM MgSO_4_ with 7:3 ratio and filtered through 0.22-μm membrane filter in the conjugation experiment. Finally, the transconjugants were selected on TSA plates supplemented with FOX (2 μg/ml) and KAN (40 μg/ml) and identified by PCR. At the same time, the negative control empty vector pLMF03 was transferred into ATCC 11845, resulting in the transconjugant ATCC 11845 (pLMF03). The MICs of tetracycline for the wild-type and transconjugants were measured as described above.

### Transfer of the *tet*(X) gene by natural transformation

The transferability of the *tet*(X) gene was verified by natural transformation as described previously (Liu et al., 2017). Briefly, the *R. anatipestifer* CH-2 genome (0.25, 0.5, 1, 2, and 5 μg) were respectively used as donor DNA and transferred into the recipient strain *R. anatipestifer* ATCC 11845. The transformants, designated ATCC 11845 [*tet*(X)], were screened using GCB plates supplemented with TET (5 μg/ml). The insertion of the transferred *tet*(X) genes was verified by PCR and sequencing. The tetracycline resistance phenotypes of transformant was determined as described above.

### Nucleotide sequence accession numbers

The sequences of *tet*(A), *tet*(B), *tet*(M), *tet*(O), *tet*(O/W/32/O), and *tet*(Q) genes in *R. anatipestifer* isolates R100, R98, R131, R96, and R159 in this study have been deposited in GenBank under the accession numbers from MF969099 to MF969104, respectively.

### Ethics statements

All animals studies were conducted in strict accordance with the recommendations of the Guide for the Care and Use of Laboratory Animals, National Research Council. The animal-use procedures were approved by the Animal Ethics Committee of the Sichuan Agricultural University (approval No. 2015-017).

## Results

### Tetracycline susceptibility of *R. anatipestifer* field isolates

**Figure 1:**
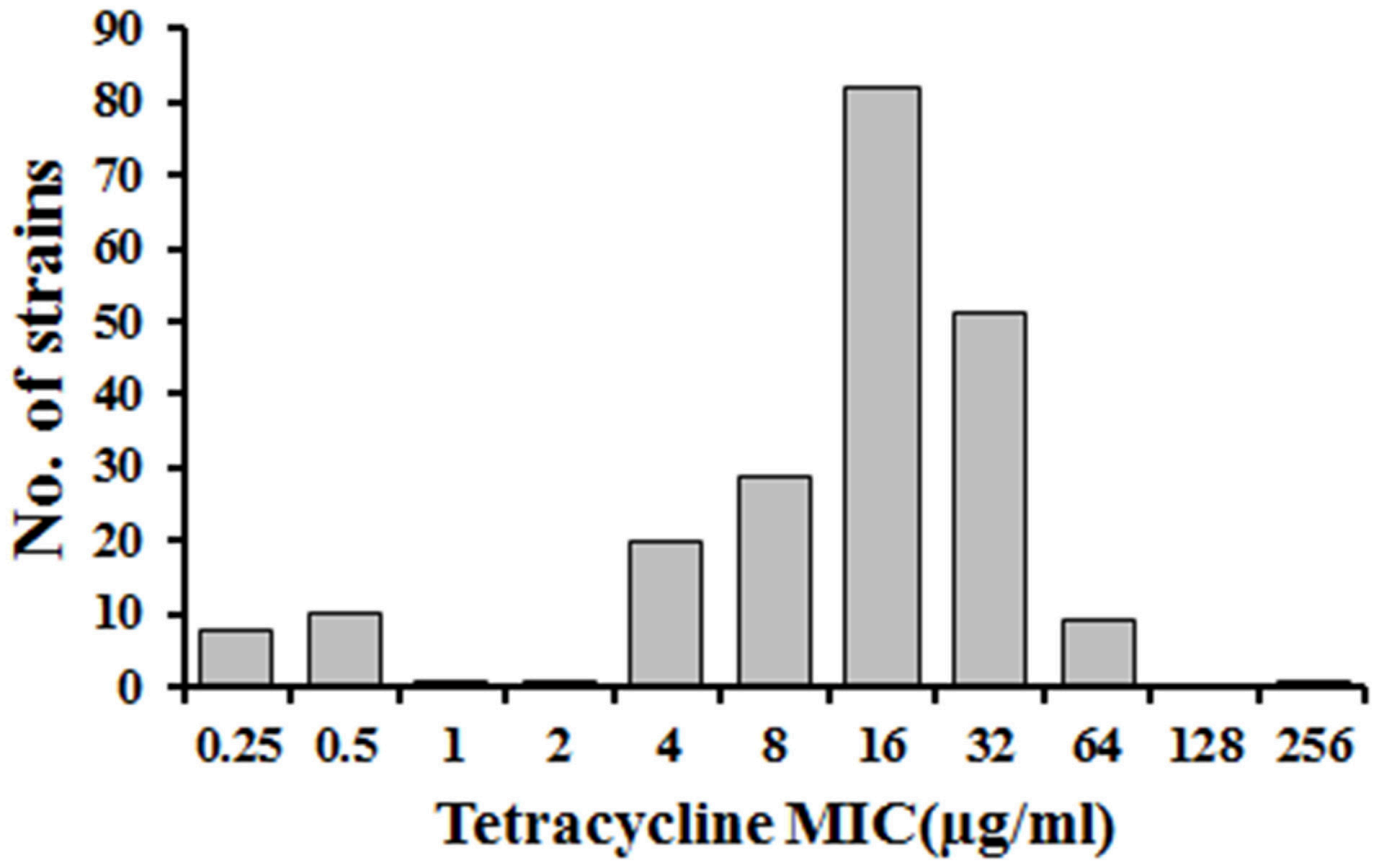
Distribution of *R. anatipestifer* field isolates with different MICs for tetracycline.

The *R. anatipestifer* field isolates were resistance to tetracycline by susceptibility test. The MICs of tetracycline in 212 *R. anatipestifer* field isolates ranged from 0.25 to 256 μg/ml (Figure 1). The results showed that 192 (90.6%) out of 212 tested strains exhibited tetracycline resistance (≥4 μg/ml), while 19 (9.0%) were susceptible (≤1 μg/ml). Only one isolate showed intermediate resistance (2 μg/ml). Most importantly, we noticed that 76.4% of isolates exhibited tetracycline resistance with MICs in range of 8–32 μg/ml.

### Prevalence of the *tet* genes in *R. anatipestifer* field isolates

To assess the prevalence of the *tet* genes in *R. anatipestifer*, the existence of these genes in 212 *R. anatipestifer* field isolates was detected by PCR and verified by sequencing in this study. The results showed that among the 12 *tet* genes, *tet*(X) was detected in 170 of 212 isolates, exhibiting the highest rate of occurrence among *tet* genes (80.2%). The detected frequency of other *tet* genes was as followed: *tet*(A) (20.8%), *tet*(M) (4.7%), *tet*(Q) (1.4%), *tet*(O) (0.9%), *tet*(B) (0.9%), and *tet*(O/W/32/O) (0.5%). However, *tet*(C), *tet*(E), *tet*(G), *tet*(K), and *tet*(W) were not detected in any of 212 tested *R. anatipestifer* field isolates (Table 2). In these positive strains, many isolates contained multiple *tet* genes simultaneously (Table 2). Namely, *tet*(X) was detected simultaneously along with *tet*(A), *tet*(M), *tet*(O), and *tet*(Q) in 31(14.6%), 5 (2.4%), 1 (0.5%), and 3 (1.4%) isolates, respectively. Two isolates (0.9%) carried *tet*(M) and *tet*(O) genes simultaneously. Moreover, one isolate (0.5%) had three commensal *tet* genes, *tet*(M), *tet*(O), and *tet*(X), respectively. The *R. anatipestifer* field isolates containing one or more *tet* genes exhibited tetracycline resistance with MICs ranging from 4 to 256 μg/ml, while no *tet* genes were detected in 19 tetracycline-susceptible isolates and 1 intermediately resistant isolate. Although several isolates carried more than one *tet* genes in this study, they did not display higher MIC values. Thus, the positive rate of genotype was in line with that of tetracycline resistance phenotype.

**Table 2:** Detection of tetracycline resistance genes in *R. anatipestifer* field isolates.

**Resistance genes**	No. of isolates positive for genes^*^	**Detected frequencies (%)**
*tet*(A)	44	20.8
*tet*(B)	2	0.9
*tet*(M)	10	4.7
*tet*(O)	2	0.9
*tet*(O/W/32/O)	1	0.5
*tet*(Q)	3	1.4
*tet*(X)	170	80.2
*tet*(A)/*tet*(X)	31	14.6
*tet*(M)/*tet*(O)	2	0.9
*tet*(M)/*tet*(X)	5	2.4
*tet*(O)/*tet*(X)	1	0.5
*tet*(Q)/*tet*(X)	3	1.4
*tet*(M)/*tet*(O)/*tet*(X)	1	0.5

* *The number of detected isolates was 212 in total*.

**Table 3:** The MICs of tetracycline and detected *tet* genes in *R. anatipestifer* isolates selected for sequencing ORFs.

***R. anatipestifer* isolates**	**MIC (μg/ml)**	**Resistance genes**						
	**Tetracycline**	***tet*(A)**	***tet*(B)**	***tet*(M)**	***tet*(O)**	***tet*(O/W/32/O)**	***tet*(Q)**	***tet*(X)**
R21^*^	4	+	–	–	–	–	–	–
R24	16	+	–	–	–	–	–	+
R66	16	+	–	–	–	–	–	+
R97	4	+	–	–	–	–	–	–
R99	4	+	–	–	–	–	–	–
R100	8	+		–	–	–	–	–
R95	8	–	+	–	–	–	–	–
R98	8	–	+	–	–	–	–	–
R133	32	–	–	+	–	–	–	–
R136	32	–	–	+	–	–	–	–
R206	256	–	–	+	–	–	–	+
R131	32	–	–	+	+	–	–	+
R132	16	–	–	+	+	–	–	–
R96	32	–	–	–	–	+	–	–
R158	16	–	–	–	–	–	+	+
R159	16	–	–	–	–	–	+	+
R218	16	–	–	–	–	–	+	+
CH−2	32	–	–	–	–	–	–	+
ATCC 11845	0.25	–	–	–	–	–	–	–

“*” *means the name of R. anatipestifer isolate*.

*“+” means the detection of tet gene was positive, while “–” means negative in R. anatipestifer strain*.

### ORF sequences analysis of the *tet* genes in *R. anatipestifer*

The tetracycline resistance pheotype and genotype of *R. anatipestifer* isolates selected for ORFs sequencing were listed in Table 3. The ORFs of the *tet*(A) gene cloned from six different *R. anatipestifer* isolates shared 99–100% identity (Table S3). The one in R100 isolate (GenBank accession no. MF969099) showed 99% sequence identity with the *tet*(A) gene from transposon Tn1721 (GenBank accession no. X61367; Allmeier et al., 1992). Two ORFs of *tet*(B) gene cloned from *R. anatipestifer* isolates showed 99% identity each other. The one in R98 isolate (GenBank accession no. MF969100) showed 100% identity with *tet*(B) gene from transposon Tn10 (GenBank accession no. J01830; Hillen and Schollmeier, 1983). The ORFs of *tet*(M) genes from five different *R. anatipestifer* isolates shared 99–100% sequence homology. The one in R133 isolate (GenBank accession no. MF969101) exhibited 96 and 95% sequence identity with *tet*(M) genes from *Streptococcus pneumoniae* (GenBank accession no. X90939; Provvedi et al., 1996), and *Enterococcus faecalis* (GenBank accession no. M85225; Su et al., 1992), respectively. Two ORFs of *tet*(O) genes cloned from *R. anatipestifer* isolates were 100% identity. The *tet*(O) gene in R131 isolate (GenBank accession no. MF969102) exhibited 99% sequence identity with the *tet*(O) genes from *Streptococcus mutans* DL5 (GenBank accession no. M20925; Leblanc et al., 1988), *Campylobacter jejuni* (GenBank accession no. M18896; Manavathu et al., 1988), and *Streptococcus pneumoniae* SA40300 (GenBank accession no. Y07780; Widdowson et al., 1996). The *tet*(O/W/32/O) from *R. anatipestifer* isolate R96 (GenBank accession no. MF969103) was a mosaic tetracycline resistance gene derived from *tet*(O) and *tet*(W) genes. Sequence alignment result showed that it had 97 and 99% sequence identity with the *tet*(W/32/O) gene from *Bifidobacterium thermophilum* (GenBank accession no. AM710601) (van-Hoek et al., 2008) and *tet*(O/W/32/O) gene from *Streptococcus suis* integrative conjugative element ICESsu32457 (GenBank accession no. FR823304) (Palmieri et al., 2012; Figure S1), respectively. The ORFs of *tet*(Q) gene from three different *R. anatipestifer* isolates shared 100% identity. The *tet*(Q) gene in R159 isolate (GenBank accession no. MF969104) exhibited 97% sequence identity with the *tet*(Q) gene from *Bacteroides thetaiotaomicron* (GenBank accession no.X58717; Nikolich et al., 1992) or *Bacteroides fragilis* 1126 (GenBank accession no. Z21523; Lépine et al., 1993).

Moreover, sequence analysis found that there were two copies of chromosomal *tet*(X) gene in *R. anatipestifer* CH-2 (Wang X. et al., 2014), which exhibited 92% sequence identity to the *tet*(X) gene previously reported on the pRA0511 plasmid in *R. anatipestifer* (Chen et al., 2010).

### MIC of tetracyclines for *R. anatipestifer* ATCC 11845 and other transconjugants

To further verify whether the identified *tet*(A), *tet*(B), *tet*(M), *tet*(O), *tet*(O/W/32/O), *tet*(Q), and *tet*(X) genes were responsible for tetracycline resistance in *R. anatipestifer*, recombinant plasmids carrying these genes amplified from different *R. anatipestifer* isolates were transferred into the tetracycline-susceptible *R. anatipestifer* ATCC 11845 by conjugative transfer. The tetracycline MICs for all transconjugants ranged from 4 to 64 μg/ml (Table 4). The transconjugants containing *tet*(A), *tet*(B), or *tet*(O) genes showed low-level tetracycline resistance, and their MIC values were 4–8 μg/ml. The transconjugants containing *tet*(M), *tet*(O/W/32/O), or *tet*(X) genes exhibited tetracycline resistance of 32 μg/ml. The tetracycline resistance of ATCC 11845 [pLMF03-R159-*tet*(Q)] was the highest among all transconjugants (the MIC value was 64 μg/ml).

**Table 4:** The MICs of tetracycline in *R. anatipestifer* transconjugants.

**Strains**	**MIC (μg/ml)**
	**Tetracycline**
ATCC 11845 (pLMF03)	0.25
ATCC 11845 [pLMF03-R66-*tet*(A)]	4
ATCC 11845 [pLMF03-R97-tet(A)]	8
ATCC 11845 [pLMF03-R99-tet(A)]	8
ATCC 11845 [pLMF03-R100-*tet*(A)]	4
ATCC 11845 [pLMF03-R95-*tet*(B)]	8
ATCC 11845 [pLMF03-R98-*tet*(B)]	8
ATCC 11845 [pLMF03-R133-*tet*(M)]	32
ATCC 11845 [pLMF03-R216-*tet*(M)]	32
ATCC 11845 [pLMF03-R96-*tet*(O/W/32/O)]	32
ATCC 11845 [pLMF03-R131-*tet*(O)]	8
ATCC 11845 [pLMF03-R159-*tet*(Q)]	64
ATCC 11845 [pLMF03-CH-2-*tet*(X)]	32
ATCC 11845 [*tet*(X)]	32

### Natural transformation

To study the transferability of the *tet*(X) gene, we transferred the *tet*(X) gene from *R. anatipestifer* CH-2 to ATCC 11845 by natural transformation. The results showed that the *tet*(X) gene could be successfully transferred into tetracycline-susceptible ATCC 11845. The maximum transformants were obtained with transformation frequency about ~10^−6^ for 5 μg *R. anatipestifer* CH-2 genome. Compared with wild-type strain ATCC11845, the transformant ATCC 11845 [*tet*(X)] exhibited 128-fold increased tetracycline resistance (from 0.25 to 32 μg/ml; Table 4). The results indicated that the *tet*(X) gene could confer tetracycline resistance and be easily transferred by natural transformation in *R. anatipestifer*.

## Discussion

Tetracyclines have been widely used for disease treatment and growth promotion in livestock (Roberts, 2003; Cheng et al., 2013). In this study, we investigated the tetracycline resistance and resistant genotypes in *R. anatipestifer* isolates in China. The results of antimicrobial susceptibility showed that tetracycline resistance in *R. anatipestifer* was widespread in China between 2011 and 2017, although the usage of this antibiotic treatment was decreased in the avian industry. By PCR detection, the genotypes of tetracycline resistance were abundant in our investigated *R. anatipestifer* isolates, including efflux genes [*tet*(A) and *tet*(B)], ribosomal protection genes [*tet*(M), *tet*(O), and *tet*(Q)], enzymatic gene *tet*(X), and mosaic tetracycline resistance gene *tet*(O/W/32/O). The total rate of positive resistance genes was as high as 90.6%. The *tet*(X) gene had the highest occurrence frequency and was the dominant mechanism conferring tetracycline resistance in *R. anatipestifer* isolates. Unexpectedly, this conclusion was in contrast to previous reports that ribosomal protection and efflux pumps were the classical tetracycline resistance mechanisms in other bacteria (Thaker et al., 2010). Meanwhile, no *tet*(C) detected in this study was also in contrast to the previous conclusion that *tet*(C) was the main mechanism of tetracycline resistance in *R*. *anatipestifer* isolates (Zhong et al., 2013). Although the positive rates were much lower than *tet*(X), *tet*(A), *tet*(B), *tet*(M), *tet*(O), *tet*(O/W/32/O), and *tet*(Q) genes were detected in *R. anatipestifer*.

Currently, single plasmid may carry multiple different *tet* genes or an isolate may contain different *tet* genes on different plasmids or some *tet* genes on plasmid and other *tet* genes in the chromosome (Roberts, 2012). For example, the *tet*(B) or *tet*(S) gene was reported to accompany with *tet*(M) gene (Kim et al., 2004; Roberts, 2012). The *E. coli* isolates from cows and pigs in slaughterhouse setting carried two different *tet* genes simultaneously [*tet*(A)/*tet*(B), *tet*(A)/*tet*(C), and *tet*(A)/*tet*(D)] (Cho and Kim, 2008). The *R. anatipestifer* isolates were found containing two different *tet* genes [*tet*(A)/*tet*(C), and *tet*(C)/*tet*(M)]. In this study, multiple *tet* genes were also found in one *R. anatipestifer* isolate, such as *tet*(A)/*tet*(X), *tet*(M)/*tet*(O), *tet*(M)/*tet*(X), *tet*(O)/*tet*(X), *tet*(Q)/*tet*(X), and *tet*(M)/*tet*(O)/*tet*(X). This phenomenon might be due to strong selective pressure and horizontal gene transfer among the various bacteria (Bryan et al., 2004).

The *tet* genes were located on conjugative, nonconjugative, and mobilizable plasmid, transposons, conjugative transposons, and *Salmonella* genomic island 1 (Roberts, 2012). In general, the efflux genes are located in chromosome, while the ribosome protection genes are often found on conjugative transposons. These mobile elements can lead to the lateral transfer of *tet* genes within and between bacteria. As no plasmid had been extracted from *R. anatipestifer* CH-2 and *R. anatipestifer* isolates R100, R98, R133, R131, R96, and R159 by several attempts using plasmid Mini kit (OMEGA), the *tet*(X), *tet*(A), *tet*(B), *tet*(M), *tet*(O), *tet*(O/W/32/O), and *tet*(Q) genes were most likely located in the chromosome of *R. anatipestifer*. We confirmed that the *tet*(X) gene could be transferred by natural transformation, and this transferability might contribute to its wide dissemination in *R. anatipestifer*.

Finally, through transferring the *tet*(A), *tet*(B), *tet*(M), *tet*(O), *tet*(O/W/32/O), *tet*(Q), and *tet*(X) genes to susceptible strain, we verified that all *tet*(A), *tet*(B), *tet*(M), *tet*(O), *tet*(O/W/32/O), *tet*(Q), and *tet*(X) genes were functional and conferred tetracycline resistance in *R. anatipestifer*. Interestingly, *tet* genes detected in this study have high identities with others reported previously, while they exhibited comparatively lower tetracycline resistance in *R. anatipestifer* (Takahashi et al., 2002; Wang W. et al., 2014). For example, *tet*(A), *tet*(B), and *tet*(O) conferred the tetracycline resistance with the MIC values from 4 to 8 μg/ml. We speculated that the low-level of resistance in *R. anatipestifer* might result from the low-level of *tet* gene expression, which needed to be further illustrated.

To the best of our knowledge, this is the first report of the presence of *tet*(A), *tet*(B), *tet*(M), *tet*(O), tet(O/W/32/O), and *tet*(Q) tetracycline resistance genes in *R. anatipestifer*. We confirmed that all the *tet*(A), *tet*(B), *tet*(M), *tet*(O), *tet*(O/W/32/O), *tet*(Q), and *tet*(X) confer the tetracycline resistance in *R. anatipestifer*. The predominant tetracycline resistance mechanism in *R. anatipestifer* is conferred by *tet*(X) gene.

## Author contributions

D-KZ, M-SW, and A-CC conceived and designed the project. H-YL, M-FL, and M-SW performed the tetracycline susceptibility test and prevalence of *tet* genes in *R. anatipestifer* isolates. D-KZ, X-XZ, and R-YJ cloned and sequenced the *tet* genes. SC, K-FS, and QY analyzed the sequences of *tet* genes. H-YL, YW, and X-YC transferred the shuttle vectors. D-KZ, H-YL, and M-SW performed the natural transformation of *tet*(X) gene. HL, D-KZ, and A-CC drafted and revised the manuscript. All authors have read and approved the final version of the manuscript.

### Conflict of interest statement

The authors declare that the research was conducted in the absence of any commercial or financial relationships that could be construed as a potential conflict of interest.

## Supplementary material

Figure S1 Sequence alignment of the tet(O/W/32/O) genes using DNAMAN 8.0 software (Lynnon-BioSoft, Ontario, Canada). GenBank accession No. annotation: MF969103, *tet*(O/W/32/O) from *R. anatipestifer* isolate R96; FR823304, *tet*(O/W/32/O) from *S. suis* integrative conjugative element ICESsu32457; AM710601, *tet*(W/32/O) from *B. thermophilum*.

Table S1 The strains and plasmids used in this study.

Table S2 Primers used in this study for PCR detection of the *tet* genes.

Table S3 The *tet* gene sequence analyses in this study.
