# Supplementary figures and images for: Various Profiles of tet Genes Addition to tet(X) in Riemerella anatipestifer Isolates From Ducks in China
Source: Front Microbiol. 2018 Mar 27;9:585. doi: 10.3389/fmicb.2018.00585 (PMC5880999; doi:10.3389/fmicb.2018.00585)

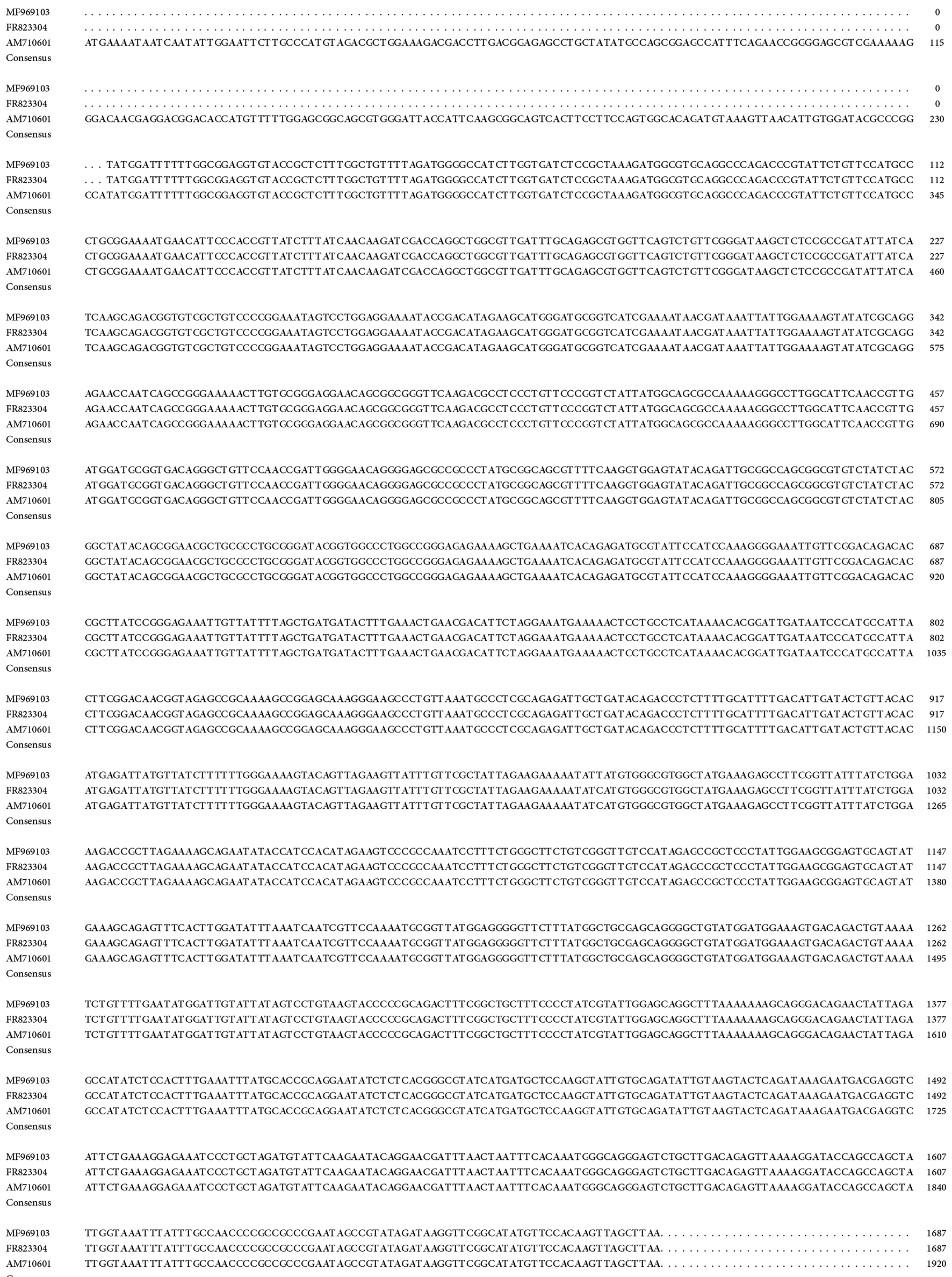

Supplement: Figure S1 — Sequence alignment of the tet(O/W/32/O) genes using DNAMAN 8.0 software (Lynnon-BioSoft, Ontario, Canada). GenBank accession No. annotation: MF969103, tet(O/W/32/O) from R. anatipestifer isolate R96; FR823304, tet(O/W/32/O) from S. suis integrative conjugative element ICESsu32457; AM710601, tet(W/32/O) from B. thermophilum. [file Image1.TIF]
