# Supplementary material for: Various Profiles of tet Genes Addition to tet(X) in Riemerella anatipestifer Isolates From Ducks in China
Source: Front Microbiol. 2018 Mar 27;9:585. doi: 10.3389/fmicb.2018.00585 (PMC5880999; doi:10.3389/fmicb.2018.00585)
Supplement: Table S1 — The strains and plasmids used in this study. [file Table1.DOCX]

### Table S1 The strains and plasmids used in this study

| **Strain or** **plasmid** | **Descriptions** | **Resource or** **references** |
| --- | --- | --- |
| **Strains** |  |  |
| *R. anatipestifer* CH-2 | Serotype 2, TET^R^, FOX^S^ | (Wang et al., 2014) |
| *R. anatipestifer* ATCC 11845 | Serotype 6, TET^S^, FOX^S^ | (Wang et al., 2012) |
| *E. coli* S17-1 | *Thi-1 thr leu ton*A *lac Y supE recA*::RP4-2-Tc::Mu KAN^R^ | Laboratory collection |
| **Plasmids** |  |  |
| pLMF03 | Shuttle vector transferred between *E. coli* and *R. anatipestifer*, AMP^R^, KAN^R^ | (Liu et al., 2016) |

TET, tetracycline; FOX, cefoxitin; KAN, kanamycin; AMP, ampicillin.
