# Supplementary material for: Various Profiles of tet Genes Addition to tet(X) in Riemerella anatipestifer Isolates From Ducks in China
Source: Front Microbiol. 2018 Mar 27;9:585. doi: 10.3389/fmicb.2018.00585 (PMC5880999; doi:10.3389/fmicb.2018.00585)
Supplement: Table S2 — Primers used in this study for PCR detection of the tet genes. [file Table2.DOCX]

### Table S2 Primers used in this study for PCR detection of the *tet* genes

| **Genes** | **Primers** | **Sequences (5’-3’)** | **Size (bp)** | **Temp (°C)** | **Reference** |
| --- | --- | --- | --- | --- | --- |
| *tet*(A) | *tet*(A)-F1 | GCTACATCCTGCTTGCCTTC | 210 | 55 | (Wu et al., 2010) |
|  | *tet*(A)-R1 | CATAGATCGCCGTGAAGAGG |  |  |  |
| *tet*(B) | *tet*(B)-F1 | GCCAGTCTTGCCAACGTTAT | 975 | 55 | (Koo and Woo, 2011) |
|  | *tet*(B)-R1 | ATAACACCGGTTGCATTGGT |  |  |  |
| *tet*(C) | *tet*(C)-F1 | CTTGAGAGCCTTCAACCCAG | 418 | 55 | (Wu et al., 2010) |
|  | *tet*(C)-R1 | ATGGTCGTCATCTACCTGCC |  |  |  |
| *tet*(E) | *tet*(E)-F1 | GTTATTACGGGAGTTTGTTGG | 212 | 55 | (Wu et al., 2010) |
|  | *tet*(E)-R1 | AATACAACACCCACACTACGC |  |  |  |
| *tet*(G) | *tet*(G)-F1 | GCTCGGTGGTATCTCTGCTC | 468 | 55 | (Wu et al., 2010) |
|  | *tet*(G)-R1 | AGCAACAGAATCGGGAACAC |  |  |  |
| *tet*(K) | *tet*(K)-F1 | TCGATAGGAACAGCAGTA | 169 | 55 | (Ng et al., 2001) |
|  | *tet*(K)-R1 | CAGCAGATCCTACTCCTT |  |  |  |
| *tet*(M) | *tet*(M)-F1 | ACAGAAAGCTTATTATATAAC | 171 | 48 | (Wu et al., 2010) |
|  | *tet*(M)-R1 | TGGCGTGTCTATGATGTTCAC |  |  |  |
| *tet*(O) | *tet*(O)-F1 | AACTTAGGCATTCTGGCTCAC | 515 | 55 | (Ng et al., 2001) |
|  | *tet*(O)-R1 | TCCCACTGTTCCATATCGTCA |  |  |  |
| *tet*(Q) | *tet*(Q)-F1 | AGAATCTGCTGTTTGCCAGTG | 167 | 48 | (Wu et al., 2010) |
|  | *tet*(Q)-R1 | CGGAGTGTCAATGATATTGCA |  |  |  |
| *tet*(W) | *tet*(W)-F1 | GAGAGCCTGCTATATGCCAGC | 168 | 60 | (Wu et al., 2010) |
|  | *tet*(W)-R1 | GGGCGTATCCACAATGTTAAC |  |  |  |
| *tet*(O/W/32/O) | *tet*(O/W)-F1 | GGAGGAAAATACCGACATA | 748 | 48 | (Palmieri et al., 2012) |
|  | *tet*(O/W)-R1 | CTCTTTCATAGCCACGCC |  |  |  |
| *tet*(X) | *tet*(X)-F1 | ATGACAATGCGAATAGATACAGAC | 1155 | 55 | (Ghosh et al., 2009) |
|  | *tet*(X)-R1 | CAATTGCTGAAACGTAAAGTC |  |  |  |
