# Supplementary material for: Various Profiles of tet Genes Addition to tet(X) in Riemerella anatipestifer Isolates From Ducks in China
Source: Front Microbiol. 2018 Mar 27;9:585. doi: 10.3389/fmicb.2018.00585 (PMC5880999; doi:10.3389/fmicb.2018.00585)
Supplement: Table S3 — The tet gene sequence analyses in this study. [file Table3.DOCX]

### Table S3 The *tet* gene sequence analyses in this study

| Sequence analysis of *tet*(A)^1^ | | | | | | | | | |
| --- | --- | --- | --- | --- | --- | --- | --- | --- | --- |
| Gene^2^ | *tet*(A)_R21 | *tet*(A)_R24 | *tet*(A)_R66 | *tet*(A)_R97 | *tet*(A)_R99 | *tet*(A)_R100 | | *tet*(A)_ Transposons Tn1721 | GenBank accession No. |
| *tet*(A)_R21 | 100% | 99% | 99% | 100% | 100% | 99% | 100% | |  |
| *tet*(A)­_R24 | 99% | 100% | 100% | 99% | 99% | 99% | 99% | |  |
| *tet*(A)_R66 | 99% | 100% | 100% | 99% | 99% | 99% | 99% | |  |
| *tet*(A)_R97 | 100% | 99% | 99% | 100% | 100% | 99% | 100% | |  |
| *tet*(A)_R99 | 100% | 99% | 99% | 100% | 100% | 99% | 100% | |  |
| *tet*(A)_R100 | 99% | 99% | 99% | 99% | 99% | 100% | 99% | | MF969099 |
| *tet*(A)_ Transposons Tn1721 | 100% | 99% | 99% | 100% | 100% | 99% | 100% | | X61367 |
| Sequence analysis of *tet*(B) gene | | | | | | | | | |
| Gene | *tet*(B)_R95 | *tet*(B)_R98 | *tet*(B)_Transposon Tn10 |  |  |  |  | |  |
| *tet*(B)_R95 | 100% | 99% | 99% |  |  |  |  | |  |
| *tet*(B)_R98 | 99% | 100% | 99% |  |  |  |  | | MF969100 |
| *tet*(B)_Transposon Tn10 | 99% | 99% | 100% |  |  |  |  | | J01830 |
| Sequence analysis of *tet*(M) gene | | | | | | | | | |
| Gene | *tet*(M)_R131 | *tet*(M)_R132 | *tet*(M)_R133 | *tet*(M)_R136 | *tet*(M)_R206 | *tet*(M)_*S. pneumoniae* | *tet*(M)_*E. faecalis* | |  |
| *tet*(M)_R131 | 100% | 100% | 99% | 99% | 100% | 96% | 95% | |  |
| *tet*(M)_R132 | 100% | 100% | 99% | 99% | 100% | 96% | 95% | |  |
| *tet*(M)_R133 | 99% | 99% | 100% | 100% | 99% | 96% | 95% | | MF969101 |
| *tet*(M)_R136 | 99% | 99% | 100% | 100% | 99% | 96% | 95% | |  |
| *tet*(M)_R206 | 100% | 100% | 99% | 99% | 100% | 96% | 95% | |  |
| *tet*(M)_*S. pneumoniae* | 96% | 96% | 96% | 96% | 96% | 100% | 96% | | X90939 |
| *tet*(M)_*E. faecalis* | 95% | 95% | 95% | 95% | 95% | 96% | 100% | | M85225 |
| Sequence analysis of *tet*(O) gene | | | | | | | | | |
| Gene | *tet*(O)_R131 | *tet*(O)_R132 | *tet*(O)_*S. mutans* | *tet*(O)_*C. jejuni* | *tet*(O)_ *S. pneumoniae* |  |  | |  |
| *tet*(O)_R131 | 100% | 100% | 99% | 99% | 99% |  |  | | MF969102 |
| *tet*(O)_R132 | 100% | 100% | 99% | 99% | 99% |  |  | |  |
| *tet*(O)_*S. mutans* | 99% | 99% | 100% | 99% | 99% |  |  | | M20925 |
| *tet*(O)_*C. jejuni* | 99% | 99% | 99% | 100% | 99% |  |  | | M18896 |
| *tet*(O)_*S. pneumoniae* | 99% | 99% | 99% | 99% | 100% |  |  | | Y07780 |
| Sequence analysis of *tet*(O/W/32/O) gene | | | | | | | | | |
| Gene | *tet*(O/W/32/O)_R96 | *tet*(W/32/O)  _*B. thermophilum* | *tet*(O/W/32/O) _*S. suis* |  |  |  |  | |  |
| *tet*(O/W/32/O) _R96 | 100% | 97% | 99% |  |  |  |  | | MF969103 |
| *tet*(W/32/O) _ *B. thermophilum* | 97% | 100% | 97% |  |  |  |  | | AM710601 |
| *tet*(O/W/32/O) _*S. suis* | 99% | 97% | 100% |  |  |  |  | | FR823304 |
| Sequence analysis of *tet*(Q) gene | | | | | | | | | |
| Gene | *tet*(Q)_R158 | *tet*(Q)_R159 | *tet*(Q)_R218 | *tet*(Q)_*B. thetaiotaomicron* | *tet*(Q)_*B. fragilis* |  |  | |  |
| *tet*(Q)_R158 | 100% | 100% | 100% | 97% | 97% |  |  | |  |
| *tet*(Q)_R159 | 100% | 100% | 100% | 97% | 97% |  |  | | MF969104 |
| *tet*(Q)_R218 | 100% | 100% | 100% | 97% | 97% |  |  | |  |
| *tet*(Q)_*B. thetaiotaomicron* | 97% | 97% | 97% | 100% | 97% |  |  | | X58717 |
| *tet*(Q)_*B. fragilis* | 97% | 97% | 97% | 97% | 100% |  |  | | Z21523 |

^1^The percentage of sequence identities were shown in this table as the result of sequence analyses.

^2^The genes were named showing their original amplification templates. For example, *tet*(A)_R21 represented that the tet(A) gene was amplified directly from the genome template of *R. anatipestifer* R21 isolate strain, *etc*.
